# Opportunities, challenges and future perspectives for target trial emulation in critical care clinical research

**DOI:** 10.1186/s13054-025-05723-x

**Published:** 2025-11-12

**Authors:** Carmen A. T. Reep, Evert-Jan Wils, Leo Heunks

**Affiliations:** 1 Department of Intensive Care, Erasmus Medical Center, Rotterdam, The Netherlands; 2 Department of Intensive Care, Franciscus Gasthuis & Vlietland, Rotterdam, The Netherlands; 3 Department of Intensive Care, Radboud University Medical Center, Nijmegen, The Netherlands

**Keywords:** Target trial emulation, Observational data, Causal inference, Critical care

## Abstract

Target trial emulation (TTE) is a powerful framework for addressing causal questions using observational data. By explicitly designing analyses to mimic a hypothetical randomized trial, TTE enables researchers to more precisely define their research questions, leading to more clinically meaningful conclusions. Its forward-looking design also helps limit common biases, such as immortal time bias and selection bias. Understanding TTE principles is essential not only for researchers working with observational data but also for clinicians who aim to critically interpret the growing number of TTE studies, as well as studies addressing causal questions without explicit use of the TTE framework.

In this review, using the timing of switch from controlled to assisted ventilation as a key example, we outline the core assumptions underpinning valid causal inference in TTE: consistency, conditional exchangeability, and positivity. We discuss practical challenges in dynamic critical care settings, including defining a meaningful time zero, handling grace periods, and selecting and properly adjusting for confounders. We also discuss caveats, such as TTE’s applicability to non-modifiable interventions, limited applicability for intention-to-treat effects, and the need for high-resolution longitudinal data. Finally, we provide a visual summary linking each trial component to key indicators of high-quality emulation.

**Supplementary Information:**

The online version contains supplementary material available at 10.1186/s13054-025-05723-x.

## Background

In critical care research, target trial emulation is increasingly used to address causal questions using observational data [1]. Familiarity with this framework is essential not only for designing future studies but also for critically interpreting existing ones. Broader understanding and application of target trial emulation can enhance methodological rigor and improve the assessment of causal validity in critical care research.

## Introduction

Questions that aim to determine whether an intervention affects an outcome of interest are known as causal questions. Answering such questions is pivotal for informing clinical decision making and can ultimately lead to improved healthcare provision [1]. The gold standard for addressing causal questions is a randomized controlled trial (RCT) [2]. Randomly assigning patients to treatment strategies and ensuring adherence allows differences in outcomes to be attributed to the intervention itself, rather than to differences in patient characteristics between groups (provided the sample size is sufficiently large) [3]. Conducting an RCT can, however, be time-consuming [2], costly [2], unfeasible or unethical [4], and environmentally impactful [5].

With the growing availability and accessibility of observational ICU-based databases [6–12], many studies strive to draw causal conclusions from these non-randomized sources. However, such studies face numerous challenges. One well-recognized challenge is the need to adjust for differences in patient characteristics between groups due to the lack of randomization. But before this, a more fundamental step is often overlooked: carefully considering what the study is truly aiming to answer. This involves explicitly specifying the treatment strategies being compared, the target population of interest, and, in the absence of randomization, defining the appropriate start of follow-up (‘time zero’). In an RCT, this process is more straightforward because the study is prospectively designed and will actually be conducted, with ethical, logistical, and safety considerations naturally guiding the study design. In contrast, when analysing observational data without the need to intervene on patients, this foundational step can be overlooked. As a result, studies may end up addressing poorly defined questions, leading to conclusions that lack clinical relevance or credibility [13, 14].

### Example of a causal question misaddressed with observational data

Consider a patient in the intensive care unit (ICU) receiving controlled invasive mechanical ventilation (IMV). The question may arise as to when to switch this patient to assisted ventilation. For instance: does switching this patient now lead to earlier successful extubation, or would it be better to wait for another day? This is a causal question, as it aims to determine whether an intervention (timing of the switch) influences an outcome (successful extubation).

Investigators attempting to address this question using observational data might compare early switching (within one day) versus delayed switching (after one day) starting follow-up at the time of IMV initiation. This would answer the question: among all patients who have just initiated IMV, is early switching more beneficial than delayed switching? However, because the timing of switch heavily depends on patient characteristics, and illness severity at intubation can vary widely, this population is highly heterogeneous. As a result, such a comparison lacks clinical relevance and is unlikely to yield meaningful or interpretable conclusions.

## Target trial emulation

An RCT addressing this question is unlikely to include all patients who have just been intubated and force an early switch regardless of clinical context, or delay switching in patients who are clearly ready (e.g., postoperative patients). Instead, it would only enrol those patients who are potentially ready to switch, for whom there is no clear evidence of harm from switching early, but also no clear evidence of harm from delaying the switch. These eligibility criteria should also be applied in the observational study, marking the start of follow-up [15]. By prospectively thinking about and explicitly specifying the components of an RCT that would answer your question (the ‘target trial’), the research question can become more clinically relevant. Emulating these target trial components using observational data is known as target trial emulation (TTE) [3]. Table 1 outlines the components of a hypothetical RCT addressing the switch timing question alongside their emulation. By including all eligible patients based on predefined criteria and following them from that point onward, common biases such as selection bias can be inherently avoided. With these foundations in place, the challenge of addressing for the lack of randomization can then be approached.

**Table 1:** Components of a hypothetical target trial addressing the switch timing question, alongside the corresponding emulation

Component	Hypothetical randomized trial	Emulation
Eligibility criteria	Inclusion: • On controlled invasive mechanical ventilation for > 2 days • Age ≥ 16 years • No neuromuscular blockers • PaO_2_/FiO_2_ ratio ≥ 150 mmHg Exclusion: • Do-not-reintubate order • Admission due to non-traumatic neurological event, traumatic brain injury, or airway protection issues	Same
Treatment strategies	**Early switch**: Switch to assisted ventilation as soon as (within one day) eligibility criteria are met for the first time, then follow usual care **Delayed switch**: Delay switching to assisted ventilation for at least one day after eligibility criteria are met for the first time, then follow usual care Switching options: • switching to an assist-only mode, • decreasing the mandatory respiratory rate, • or reducing sedation	Same, with switch timing identified by changes in ventilation mode or respiratory rate compared to the previous measurement
Assignment procedures	Participants are randomly assigned to a strategy at the time they become eligible for switching	Patients are assigned to both strategies at eligibility and are artificially censored once they deviate from the assigned strategy.
Follow-up period	From randomization until successful extubation, ICU discharge, death, loss to follow-up, or 28 days, whichever occurs first	From eligibility until artificial censoring, successful extubation, ICU discharge, death, loss to follow-up, or 28 days, whichever occurs first
Outcome	Successful extubation: extubation without reintubation or death within 7 days, or ICU discharge without invasive ventilation within 7 days, whichever comes first	Same
Causal contrasts of interest	Per protocol effect	Same
Analysis plan	Compare 28-day cumulative incidence of successful extubation between strategies, accounting for competing risks of ICU death and discharge	Same, with weights to balance time-varying confounders between patients adhering to the strategy and those censored

When well-executed using sufficiently rich observational databases, TTE can generate real-world evidence without the strict controlled setting of RCTs [16], offer timely insights in urgent situations (such as a pandemic), and guide the design of promising future RCTs. As TTE is increasingly used in critical care research [1, 17–25], familiarity with this framework is essential not only for researchers conducting studies, but also for clinicians and reviewers to assess the validity of causal claims. Understanding TTE principles also supports a more critical examination of studies that do not use the framework but still aim to answer causal questions, as it helps identify potential sources of bias. In the following sections, we explain the TTE framework and illustrate its application using the switch timing question.

### Three key assumptions

A TTE can be considered valid if three key assumptions are met [26]. The first is *consistency*, which requires the intervention to be sufficiently well-defined, capturing all aspects that, if varied, would lead to different outcomes [27]. In the switch example, if the switch can occur through different strategies, such as switching to assist-only mode, reducing the set respiratory rate, and/or decreasing sedation, each strategy should yield comparable outcomes (or at least not opposing ones). This allows estimation of the effect of switching, averaged across the different strategies used [28]. Well-defined strategies could be framed as ‘switch to assisted ventilation as soon as (within one day) eligibility criteria are met for the first time, via any of the above strategies, followed by usual care,’ versus ‘delay the switch for at least one day after first meeting eligibility criteria, via any of the same methods, followed by usual care’ [15].

If the effects of the different strategies would differ meaningfully, the intervention should be defined more narrowly, for example by focusing on a single switching method. Whether consistency holds should be judged by expert knowledge [27] or assessed through sensitivity analyses (e.g., analysing each version of the intervention separately [15]).

Secondly, the *conditional exchangeability* assumption must hold, meaning that after adjusting for confounders, patients in different intervention groups should be comparable (as would be achieved by randomization in an RCT). Confounders are variables that must be accounted for to estimate the true effect of a treatment. A practical approach is to adjust for all pre-treatment variables that influence treatment decisions and independently predict the outcome. For example, in the switch scenario, sedation level should be adjusted for, as higher sedation affects switch timing (being associated with delayed switching decisions) and is also linked to longer IMV duration within an intervention group. When such common causes of both treatment and outcome are unavailable, proxies may be used to reduce resulting bias. For instance, cardiovascular instability may influence both switching decisions and IMV duration through various complications but is not directly measurable as a single factor. A composite score, such as the cardiovascular component of the SOFA score, can serve as an approximate measure.

Adjusting for variables that influence only the outcome can improve precision. For example, age may not typically affect the decision on switch timing but is associated with the outcome, making it appropriate to include in adjustment. In contrast, adjusting for factors that affect only intervention decision and are entirely unrelated to the outcome except through the intervention (i.e., instrumental variables) should be avoided, as this can amplify bias from unmeasured confounding [29]. However, true instrumental variables are rare in practice. Additionally, variables caused by the intervention that subsequently affect the outcome should not be controlled for. For example, muscle weakness resulting from a delayed switch, which may reduce tolerance to spontaneous breathing trials and thereby delay extubation.

**Fig. 1:**
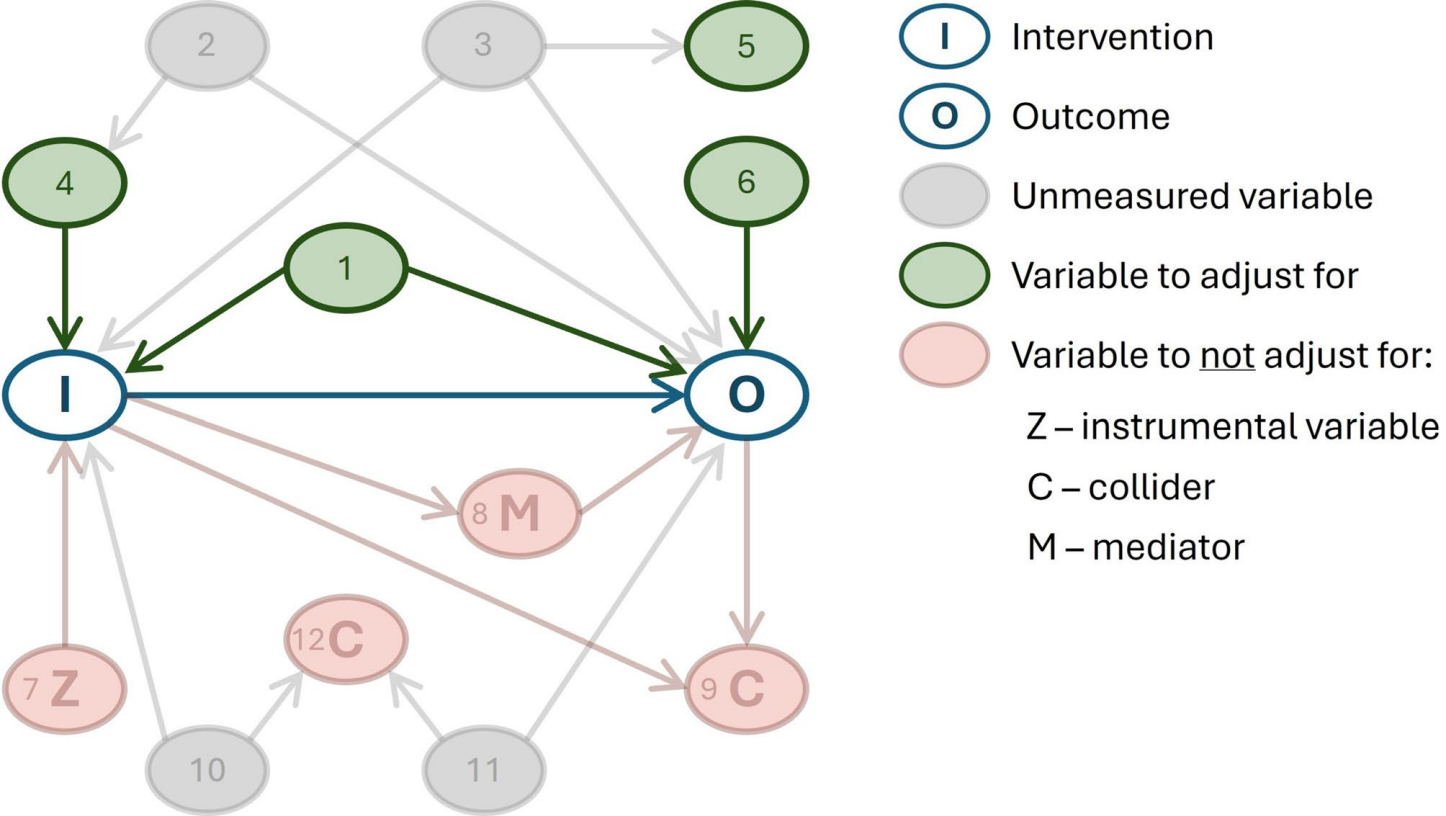
Directed acyclic graph (DAG) showing variables to adjust for and not to adjust for. This figure presents a directed acyclic graph (DAG), where arrows represent causal relationships and nodes represent variables. To estimate the causal effect of the intervention (I) on the outcome (O), confounding variables must be accounted for. A practical approach is to adjust for all pre-intervention common causes of both intervention and outcome (node 1). When these are unavailable (nodes 2, 3), suitable proxies can help reduce bias (nodes 4, 5). Variables that affect only the outcome may also be adjusted for to improve precision (node 6). In contrast, adjustment should be avoided for: (a) variables linked to the intervention that are entirely unrelated to the outcome except through the intervention (instrumental variables, node 7); (b) variables caused by the intervention that affect the outcome (mediators, node 8); and (c) variables caused by both the intervention and the outcome (colliders, node 9), or caused by factors separately linked to both intervention (node 10) and outcome (node 11) (colliders, node 12). A DAG is worked out for the switch example in the supplements (Figure E1). *DAGitty* is a useful online tool for drawing DAGs and assessing appropriate adjustment variables [46]

Directed acyclic graphs (DAGs) can help structure and visualise this causal reasoning and should be guided by literature and expert input [30]. Figure 1 shows a DAG providing an overview of which variables to adjust for and which not to, while Figure E1 presents an example DAG for the switch example. Although the absence of residual confounding cannot be confirmed, its potential impact can be evaluated through sensitivity analyses. For example, the E-value, which quantifies how strong an unmeasured confounder would need to be to fully explain away the observed effect [17, 21, 31].

The third assumption, the *positivity* assumption, requires that every eligible patient should have had the possibility of receiving all interventions. In the switch example, if no deeply sedated patients undergo early switching, and sedation level also influences the outcome of interest, confounder balance between groups cannot be achieved. In such cases, observed differences in outcomes may reflect variations in sedation practices rather than the timing of the switch. Violations of this assumption can be detected by limited overlap in confounder distributions across groups or, in weighting methods, by extreme weights. These violations often arise when only few patients follow the treatment strategy under study. Solutions include refining strategies to better reflect clinical practice or excluding subgroups that never receive the intervention.

### Challenges and recommendations

Beyond these three assumptions, TTE presents additional challenges. Below, we highlight the most important ones and provide recommendations for addressing them.

**Fig. 2:**
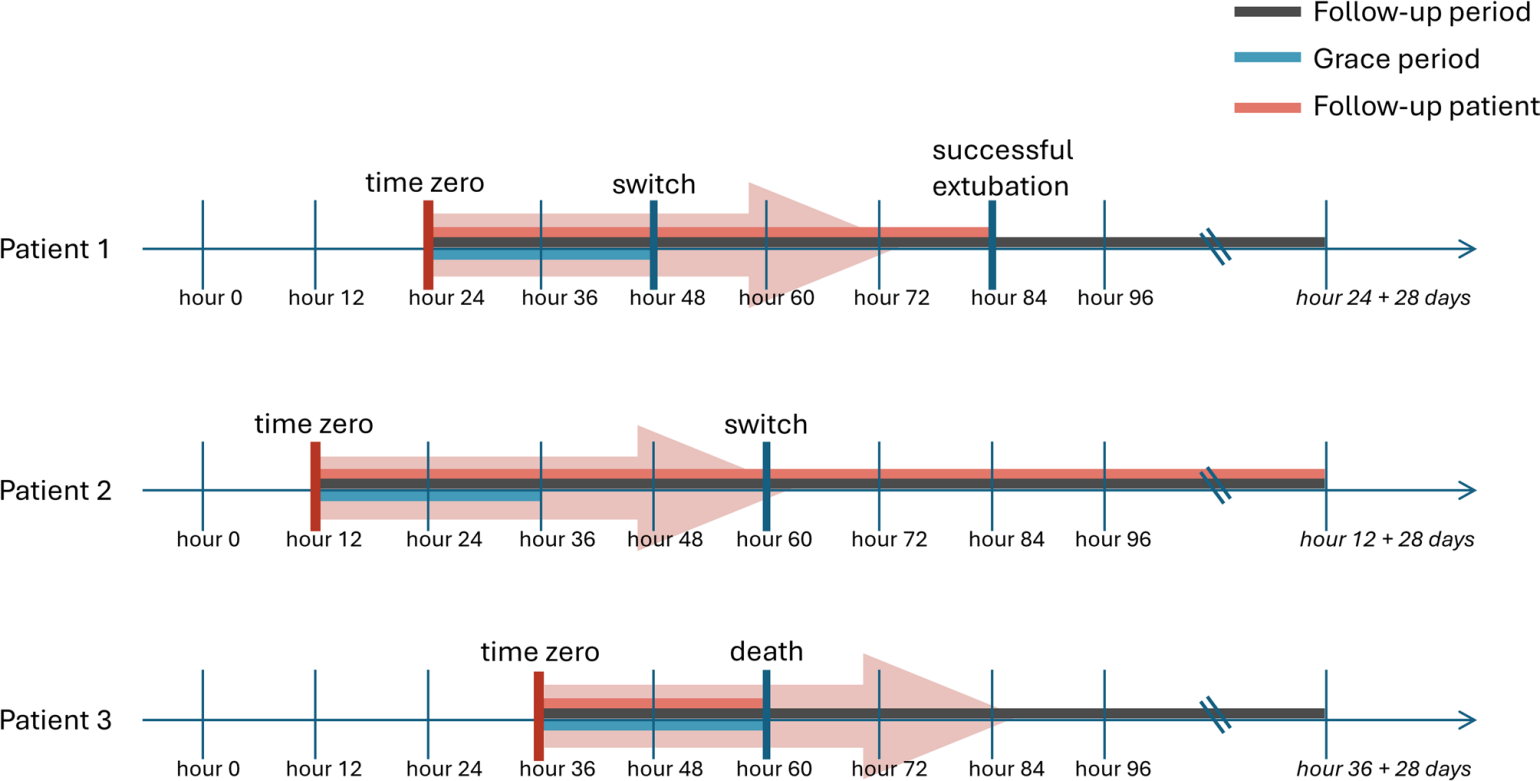
Illustration of the forward-looking approach to observational data using the switch example.This figure illustrates how the switch timing question is tackled prospectively using observational data collected every 12 hours. The treatment of interest is the switch, which is a point intervention. Outcomes (successful extubation or death) and any switches occurring between data collection points are captured at the next collection point. Follow-up begins at the moment a patient meets pre-specified eligibility criteria (time zero) and extends for 28 days. Two treatment strategies are compared: (1) early switching – within 24 hours of eligibility, and (2) delayed switching – after 24 hours, with this 24-hour window referred to as the ‘grace period’. Patient 1 meets eligibility criteria at hour 24, switches within the next 24 hours (within the grace period), so is said to have followed the early switch strategy. This patient is followed up until reaching the outcome of interest 60 hours after time zero

First, correctly defining time zero (the moment eligibility criteria are met) is crucial but challenging. Time zero should always precede or coincide with treatment initiation to avoid comparing non-users to prevalent users. Otherwise, selection bias occurs because prevalent users have, by definition, already survived a period under treatment [32]. Additionally, eligibility should not rely on ‘future information’: it is important to clearly define time zero and think forward from that point onward, rather than looking back from outcomes or excluding patients based on information not yet available at time zero (Fig. 2). For example, when investigating the optimal timing of switching, if the target population is patients who have received IMV for at least a certain duration (e.g., two days), this should be a predefined inclusion criterion, with time zero set after that duration is reached. Another challenge with defining time zero is that patients may meet eligibility criteria at multiple time points, making it unclear which should be chosen as time zero. For intervention efficacy questions comparing initiating an intervention to never initiating (such as whether a switch should be made in a subgroup of patients with a baseline P/F ratio between 150 and 200), time zero can be defined as the first eligible moment, a randomly selected one, or all eligible moments. The latter approach is known as a sequential trials approach [33, 34], and allows for more efficient use of available data. In contrast, for questions about intervention timing (given all patients eventually switch, determining whether switching early improves outcomes), time zero must be the first moment the patient becomes eligible [35].

The next challenge involves treatment strategies that include a grace period: a pre-defined time window between meeting eligibility criteria and the actual treatment initiation [36]. Some strategies incorporate a grace period by design; for example, early switching allows for switches within one day after time zero. In other cases, incorporating a grace period makes the treatment strategy more realistic by accounting for delays between assignment and initiation, thereby reflecting actual clinical practice (thus capturing a larger group of patients) [36]. The challenge with grace periods, however, is dealing with outcomes occurring in this grace period. In an RCT, outcomes during this period would be counted towards the assigned arm. In observational data studies, a common mistake is counting outcomes (e.g., death) during this grace period towards the ‘no intervention’ (delayed switch) arm. This makes the intervention (early switch) inadvertently appear more beneficial, which is known as immortal time bias [37]. This bias can be addressed by either randomly assigning outcomes during the grace period to a treatment arm or by cloning eligible patients and assigning outcomes to all treatment strategies their data remained compatible with [36].

A third challenge of TTE is proper adjustment for confounding, which depends on the specific treatment strategy being emulated (Table 2). For static point interventions without a grace period (e.g., a single dose of medication at baseline versus none), traditional methods like stratification or propensity score matching may suffice [26]. However, most causal questions involve more complex scenarios where the intervention does not occur at time zero or varies over time. Examples include strategies with a grace period, sustained interventions such as “switch to assisted ventilation and maintain on it as long as P/F ratio remains ≥ 150 mmHg” [21], or dynamic treatment regimes in which treatment is initiated only when a time-varying clinical threshold is crossed [24]. For instance, switch to assisted ventilation as soon as P/F ratio rises above 150 mmHg vs. switch as soon as P/F rises above 200 mmHg. For these time-varying scenarios, conditional exchangeability and positivity must hold at each decision point, conditional on prior treatment and covariate history [38]. Appropriately addressing time-varying confounding in these settings requires advanced analytic approaches, known as G-methods (e.g., inverse-probability-of-treatment weighting, the clone-censor-weight design, the parametric g-formula, and g-estimation) [26, 38, 39]. To support broader adoption of such analytical approaches, more open-access code templates should be developed and shared.

**Table 2:** Different treatment strategies using the switch example

Category	Type	Description	Example
Strategy	Static	Intervention is assigned at baseline	Switch to assisted ventilation within 1 day of eligibility vs. after 1 day
	Dynamic	Intervention depends on evolving patient characteristics	Switch to assisted ventilation as soon as P/F rises above 150 vs. switch as soon as P/F rises above 200 mmHg
Intervention	Point	Once at a specific time point	Switch to assisted ventilation
	Sustained	Continuous throughout follow-up	Switch to assisted ventilation and maintain on it as long as P/F ratio remains ≥ 150 mmHg.
Grace period	Present	Intervention is allowed within a predefined time window following eligibility, or upon reaching a threshold	Switch to assisted ventilation within 24 h of eligibility
	Absent	Intervention must occur exactly at time of eligibility or upon reaching a threshold	Switch to assisted ventilation immediately when P/F rises exceeds 150 mmHg

P/F ratio = PaO_2_/FiO_2_ ratio

### Caveats

Although target trial emulation provides a structured approach to causal inference, practical constraints may limit its applicability. We describe some caveats below.

First, comprehensive longitudinal data with sufficient granularity are essential to define a meaningful time zero, assess adherence to treatment strategies, adjust for time-varying confounders, and capture relevant outcomes. In critical care, granularity is particularly challenging due to the highly dynamic clinical course of patients. Confounders must be measured shortly before the intervention for proper adjustment, and key events, such as switches between recorded intervals, should not be missed. When interventions and confounders are recorded within the same time interval, care must be taken to ensure confounders are measured before the intervention, as variables recorded afterward may already be influenced by treatment. In prospective observational studies, ideally the intended research questions should guide data collection. Otherwise, rich electronic health record datasets offer a valuable resource for target trial emulation.

Second, while the TTE framework is not restricted to feasible or practical interventions, there is ongoing debate about the interpretability for non-modifiable interventions [27]. For example, pulse oximetry devices estimate arterial oxygen saturation from the relative absorbance of two wavelengths of light. It is hypothesized that such devices may be less accurate in patients with darker skin tones, as pigmentation can affect light absorption [40]. One could technically apply the TTE framework by treating skin tone as the “intervention” (e.g., comparing Fitzpatrick Skin Type >IV versus ≤ III [41]) and estimating its causal effect on hidden hypoxemia. However, because skin tone is not modifiable, such findings do not directly guide decision-making (one cannot alter skin tone as a means to prevent hidden hypoxemia). Infeasible interventions like this can highlight potential targets for action [27], such as avoiding reliance on pulse oximeters for patients with darker skin or improving device technology to limit racial disparities, but studying modifiable interventions in a TTE is generally most informative. Of note, while skin tone can be considered an appropriate “intervention” for pulse oximetry accuracy, it is not well-defined as a proxy for race on health or income, where the relevant intervention would reflect social, historical, biological, and environmental factors rather than actual skin color [27].

Lastly, RCTs often estimate intention-to-treat effects, which are considered unbiased estimates when most patients stick to their assigned strategies. However, in observational studies, the absence of random assignment means there is no inherent intention to follow a specific treatment strategy, making non-adherence more likely [26]. For this reason, per-protocol effects are usually more informative in target trial emulation, as they estimate the effect among patients who actually adhered to the strategy. The intention-to-treat effect, or more precisely its *observational analog* (since there is no actual intent), is generally only appropriate for strategies without a grace period and no opportunity for non-adherence after starting the intervention, such as point interventions or sustained strategies like “start treatment, then follow usual care” [26].

**Fig. 3:**
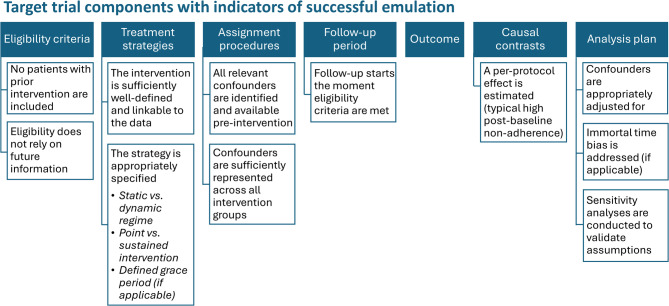
Target trial components with indicators of successful emulation. Components of the target trial with indicators for successful emulation

Figure 3 summarizes the target trial components with indicators of successful emulation based on the key assumptions, challenges and considerations.

## Discussion

By framing observational data analyses as hypothetical trials and adopting a forward-looking analytical approach, researchers can better clarify their research questions, limit common biases, and draw clinically meaningful conclusions. This is known as the target trial emulation (TTE) framework. This review uses a critical care example to illustrate the assumptions, challenges, and considerations of the TTE framework. Recognizing the key features of well-conducted TTE studies supports researchers in designing and executing such studies, while at the same time helps clinicians and reviewers critically assess the credibility of causal claims.

The use of TTE is especially valuable in critical care research due to the data-rich environment and the dynamic nature of patient conditions and treatment decisions, resulting in high-granularity databases [1]. It enables the timely evaluation of urgent clinical questions, such as those arising during pandemics, without the delays associated with conducting RCTs. In addition, TTE may guide the design of costly and resource-intensive RCTs, potentially reducing the risk of negative trials in which no treatment effect is observed.

While the TTE framework enhances the ability to draw causal inferences from observational data, it depends heavily on correct application and data quality. A key limitation is that unmeasured confounding can never be fully ruled out, meaning strong causal claims remain tentative. Nonetheless, studies have shown that when observational data analyses closely emulate actual RCTs and are well-conducted, the resulting conclusions often closely align [42].

When more ICU studies using observational data adopt the TTE framework, the quality of evidence is likely to improve, ultimately enabling clinicians to make better-informed decisions in complex and rapidly evolving care environments.

Future work should focus on wider dissemination of TTE principles among clinical researchers, including the integration of the framework into educational programs. Sharing diverse methodological approaches for achieving confounding balance over time, along with open-access code templates, would greatly support adoption. Examples include the work by Morzywołek and colleagues on dynamic treatment regimes [43], and by Maringe and colleagues on evaluating the benefit of surgery for elderly lung cancer patients with a six-month grace period [36]. Additionally, the development of standardized reporting checklists or guidelines for TTE studies is needed to improve transparency and consistency, such as the TARGET guideline [44]. Finally, since TTE studies can offer stronger causal evidence than conventional observational studies, the GRADE framework for assessing quality of evidence [45] should consider upgrading the rating of evidence derived from TTE studies above that of standard observational designs.

## Conclusions

In conclusion, when addressing causal questions with observational data, framing the analysis as a hypothetical RCT helps formulate clinically meaningful questions and reduce biases. Recognizing and applying key TTE principles can improve the design and interpretation of ICU research.

## Supplementary Information

Supplementary Material 1

## Data Availability

No datasets were generated or analysed during the current study.

## Abbreviations

TTE: Target trial emulation
ICU: Intensive care unit
IMV: Invasive mechanical ventilation
RCT: Randomized controlled trial
SOFA: Sequential organ failure assessment
