# Supplementary material for: Opportunities, challenges and future perspectives for target trial emulation in critical care clinical research
Source: Crit Care. 2025 Nov 12;29:484. doi: 10.1186/s13054-025-05723-x (PMC12613904; doi:10.1186/s13054-025-05723-x)
Supplement: Supplementary file 1 — Supplementary Material 1 [file 13054_2025_5723_MOESM1_ESM.docx]

**Opportunities, challenges and future perspectives for target trial emulation in critical care clinical research**

*Carmen A.T. Reep, Evert-Jan Wils, Leo Heunks*

Supplemental materials

**Figure E1. Directed acyclic graph (DAG) for the switch scenario**

**
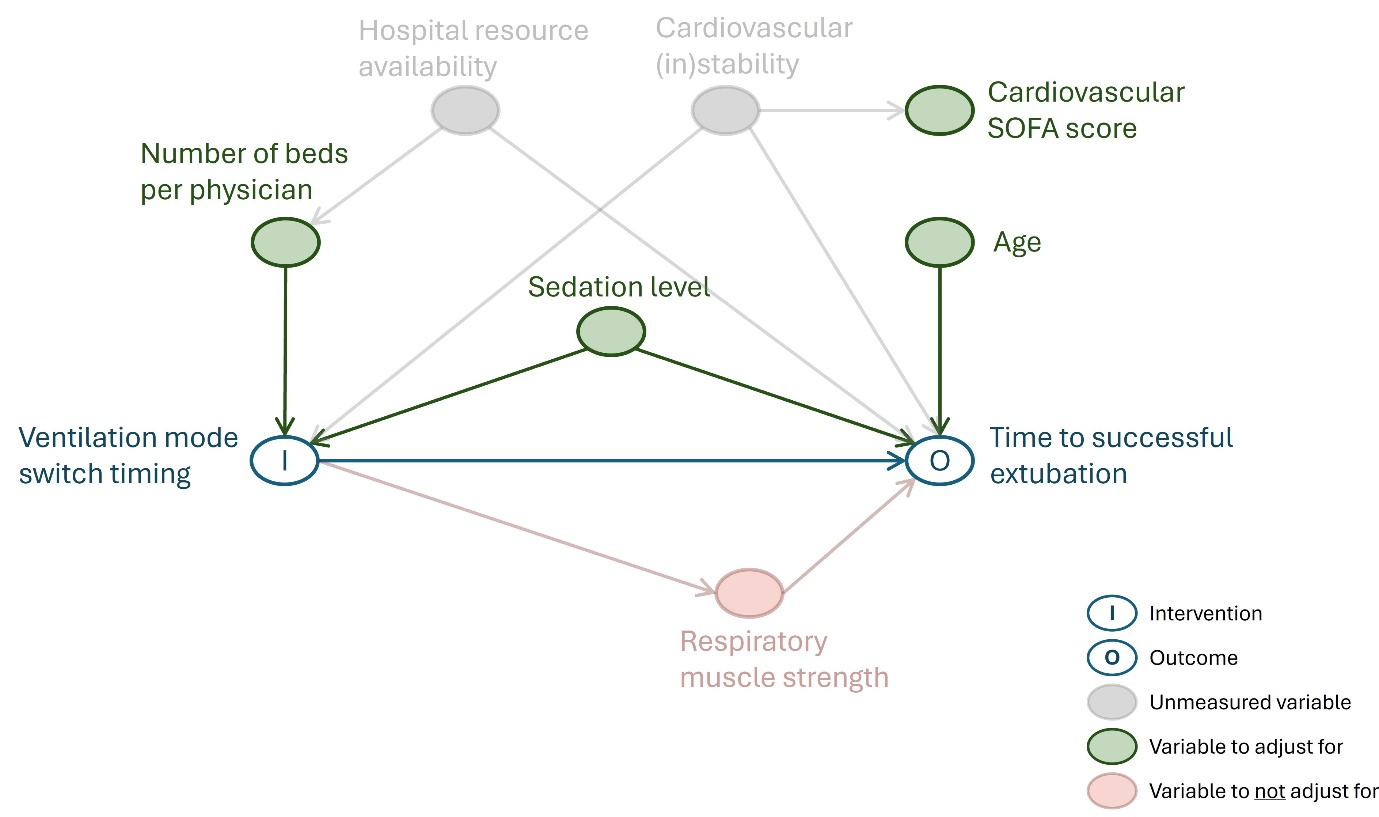
**

*Figure E1 caption.* This figure presents a directed acyclic graph (DAG), where arrows represent causal relationships and nodes represent variables. To estimate the causal effect of the intervention (I; ventilator switch timing) on the outcome (O; time to successful extubation), confounding variables must be accounted for. For example, sedation level should be accounted for because it influences both switch timing (higher sedation delays switching) and IMV duration (within an intervention group, higher sedation is linked to longer IMV). When common caused of both intervention and outcome are unavailable, proxies can help reduce bias (e.g., cardiovascular SOFA score or number of beds per physician) (1).

Variables that affect only the outcome, such as age, can be adjusted for to improve precision. In contrast, variables caused by the intervention and lying on the causal pathway to the outcome should not be controlled for (1,2). For example, muscle weakness resulting from a delayed switch may reduce tolerance to spontaneous breathing trials, thereby delaying extubation.

*DAGitty* is a useful online tool for drawing DAGs and assessing appropriate adjustment variables (3).

**References**

1. VanderWeele TJ. Principles of confounder selection. Eur J Epidemiol. 2019 Mar 15;34(3):211.

2. Tennant PWG, Murray EJ, Arnold KF, Berrie L, Fox MP, Gadd SC, et al. Use of directed acyclic graphs (DAGs) to identify confounders in applied health research: review and recommendations. Int J Epidemiol. 2021 Apr 1;50(2):620–32.

3. Textor J. Drawing and Analyzing Causal DAGs with DAGitty. 2023; Available at: <https://www.dagitty.net/dags.html>
